# Evaluation of visual and optical quality following phacoemulsification cataract surgery with diffractive multifocal intraocular lens implantation: An observational study

**DOI:** 10.1097/MD.0000000000038905

**Published:** 2024-07-19

**Authors:** Daoguang Wang, Yuanxiao Ma, Tingting Hu, Yucheng Wang, Keli Cai

**Affiliations:** a Department of Cataract, Huaxia Eye Hospital Group, Jinan Huashi Eye Hospital, Jinan, Shandong Province, China; b Department of Cataract, Jinan Huashi Eye Hospital, Jinan, Shandong Province, China.

**Keywords:** cataract phacoemulsification, modulation transfer function, pupil size, scatter light values, Tecnis ZMB00 MIOL, visual quality, wavefront aberrations

## Abstract

The assessment of patient satisfaction following cataract surgery is heavily reliant on the evaluation of visual quality, specifically after the placement of diffractive multifocal intraocular lenses (MIOLs) under varying pupil conditions. The objective of this study was to examine the visual and optical clarity following cataract phacoemulsification and the use of Tecnis ZMB00 MIOL for implantation. The study involved 116 individuals (135 eyes) who received cataract phacoemulsification and underwent Tecnis ZMB00 MIOL implantation. Assessments were conducted 1 week and 3 months after the surgery. These assessments involved measuring uncorrected and corrected visual acuity for distant, intermediate, and near vision. Additionally, scatter light values and wavefront aberrations were measured under different aperture settings of 3 and 5 mm. There was no noticeable disparity in visual acuity between 1 week and 3 months after the surgery. After 3 months of surgery, there was a considerable decrease in scatter light values and spherical aberrations compared to the values observed 1 week after surgery, under the setting of a 5 mm aperture. Moreover, the modulation transfer function values showed a significant rise after 3 months following the surgery, particularly under the 5 mm aperture condition. The most substantial increase was observed at the intermediate spatial frequency of 20 cycles per degree (cpd), in comparison to the values obtained 1 week after the operation. The combination of cataract phacoemulsification and Tecnis ZMB00 MIOL implantation yielded favorable visual acuity at various distances for patients. Furthermore, enhancements in the measurements of scattered light, higher-order aberrations, and modulation transfer function values were noted 3 months after the surgical procedure, specifically under the condition of a 5 mm pupil. These findings suggest an increase in visual clarity and night vision to a certain degree.

## 1. Introduction

With the ongoing expansion of the global elderly population, there is a projected significant rise in the occurrence of vision-related disorders, particularly cataracts. The conventional treatment for this condition entails the surgical extraction of the damaged lens and its substitution with a monofocal intraocular lens (IOL). Advancements in technology and surgical procedures have facilitated the creation and utilization of multifocal intraocular lenses (IOLs), which provide the potential for comprehensive visual clarity and the ability to see clearly at different distances.^[1]^ The growing need for superior vision and enhanced visual comfort after refractive cataract surgery has resulted in a broader emphasis that goes beyond only restoring visual acuity. In order to provide modern patient-centered care, it is necessary to prioritize the enhancement of visual quality in various viewing settings and lighting environments. Nevertheless, multifocal IOLs have been linked to specific visual problems after surgery, specifically glare, and halos.^[2]^ These abnormalities, often seen during the night, present considerable difficulties for patients’ overall well-being and can last for a period of 2 months after surgery.^[3,4]^

Pupil size, which can vary due to light circumstances and individual variances, is a possible issue that can impact the visual quality of multifocal IOLs. While a smaller pupil size can enhance visual quality by reducing higher-order aberrations, it can also hinder the advantages of multifocal IOLs and disrupt the equilibrium between distance and close vision. Nevertheless, the specific impact of various pupil circumstances on the visual clarity of diffractive multifocal IOLs has not been thoroughly studied.^[5,6]^ In addition, quantitative assessments such as scattered light, wavefront aberrations, and the modulation transfer function (MTF) values offer valuable insights into the optical performance of the visual system. However, these measures have not been extensively investigated in relation to diffractive multifocal IOLs under various pupil conditions.^[7]^ Crucially, there has been insufficient investigation into the variations of these characteristics over time and their associations with subjective visual quality and the ability to adjust to new visual settings.

Therefore, the objective of this study is to address the noted deficiencies in the current body of research. We conducted a longitudinal study to assess the subjective and objective visual quality following the implantation of full-optical diffractive aspheric multifocal IOLs. We conducted an evaluation that included measuring visual acuity at different distances, both close and far, after the surgery. We also measured the amount of scattered light, wavefront aberrations, and MTF values for different sizes of the pupil. By comprehensively studying the impact of different pupil conditions on visual quality, our goal was to improve the individualized approach to patient care. This would provide clinicians with relevant information, enabling them to make better educated judgments about IOL selection. In addition, our goal is to enhance patient satisfaction and visual comfort after multifocal IOL implantation by providing patients with a more precise understanding of the expected outcomes. Although our study primarily examined the performance of Tecnis ZMB00 MIOLs, the findings can be used to further understand the performance of multifocal IOLs, particularly when compared to existing literature on monofocal IOLs. The findings of our study could provide valuable insights for improving multifocal IOL optimization procedures. However, in order to draw more conclusive conclusions about broader developments in IOL technology, it would be important to directly compare these results with those obtained from monofocal IOLs.

## 2. Methods

### 2.1. Study population and selection criteria

This study retrospectively observed a cohort of 116 patients (135 eyes) who received cataract phacoemulsification and had Tecnis full-optical diffractive aspheric multifocal intraocular lenses (Tecnis ZMB00 MIOL, J&J, New Brunswick) implanted at our hospital from September 2020 to July 2022. The sample consisted of 67 males (83 eyes) and 49 females (52 eyes), with a mean age of 62 ± 16 years. The Tecnis ZMB00 MIOL is characterized by its proprietary diffractive design which enhances multifocal visual performance. This lens is specifically designed to improve visual acuity at multiple distances by distributing light to distant and near focal points based on the pupil size, and its aspheric surface reduces spherical aberrations. The eligibility criteria consisted of corneal astigmatism < 1.5 diopters, potential acuity measurement of at least 0.5, pupil diameter > 2.5 mm under normal lighting conditions, Kappa angle < 0.5 mm, and a signed informed consent for both the operation and participation in the study. Subjects were recruited considering a diverse demographic to minimize selection bias and ensure broad applicability of the study results. The exclusion criteria encompassed other ocular conditions and prior ocular procedures. The study protocol received approval from the Ethics Committee of Jinan Huashi Eye Hospital.

### 2.2. Surgical procedure

The procedures were conducted with topical anesthetic by a skilled surgeon utilizing the Infiniti phacoemulsification system (Alcon, Fort Worth). The Tecnis ZMB00 MIOL was consistently positioned in the central region of the capsular bag in all instances. The IOL power was determined using the Holladay II formula, with a target refraction range of +0.00 to +0.25 D. This measurement was performed using the IOL Master 700, manufactured by Carl Zeiss in Germany.

### 2.3. Assessment of visual and optical quality

#### 2.3.1. Visual acuity and optical measurements

Visual acuity and optical measurements were conducted at 2 specific postoperative time points: 1 week after surgery and 3 months after surgery.

#### 2.3.2. One *week after surgery*

This early assessment aids in capturing the initial visual recovery and optical quality after lens implantation. Immediate postoperative results can provide insights into surgical precision, early refractive outcomes, and potential immediate complications.

#### 2.3.3. Three months after surgery

This period allows for stabilization of refractive outcomes and captures any adaptive neural changes that can influence visual performance. The 3-month mark is considered a standard in the ophthalmic literature to evaluate the stable visual and refractive results after intraocular surgeries.

Visual acuity measurements included uncorrected distance visual acuity (UCDVA), best-corrected distance visual acuity (BCDVA), uncorrected intermediate visual acuity (UCIVA), distance-corrected intermediate visual acuity (DCIVA), uncorrected near visual acuity (UCNVA), and distance-corrected near visual acuity (DCNVA). All visual acuity measurements were recorded using the LogMAR scale.

#### 2.3.4. Scattered light

Scattered light was assessed at both 1 week and 3 months post-surgery using a C-Quant stray light meter (Oculus, Germany). The eyes of each patient were fully dilated using tropicamide eye drops (Santen, Japan), and the scattered light values were measured under different aperture conditions with 3 and 5 mm pinholes.

#### 2.3.5. Postoperative aberrations

Wavefront aberrations were also assessed at 1 week and 3 months post-surgery using the iTrace Visual Function Analyzer (Tracey Technologies, USA). Both WF and CT modes were automatically detected to compute the total and higher-order aberrations, spherical aberration, coma, trefoil, and MTF values at various spatial frequencies under 3 and 5 mm aperture conditions.

### 2.4. Statistical analyses

The data gathered, which included postoperative visual acuity, scattered light values, wavefront aberrations, and MTF values, were meticulously analyzed using the Statistical Package for Social Sciences (SPSS) version 22.2. The first step was to determine the distribution of the acquired data. The Shapiro–Wilk test, a robust method for evaluating the normality of data, was employed for this objective. Nonapproaches were employed for comparative purposes when the data deviated from a normal distribution. The Mann–Whitney *U* test was used to compare differences between 2 independent groups. This test is suitable for situations when the dependent variable is either ordinal or continuous, but not regularly distributed. The threshold for statistical significance was established at a *P* value below .05.

## 3. Results

### 3.1. Postoperative visual acuity

As shown in Table 1, there were no statistically significant differences in uncorrected and corrected distant, intermediate, and near visual acuity at 1 week and 3 months postoperatively (all *P* > .05). Specifically, the uncorrected distance visual acuity (UCDVA) slightly decreased from 0.06 ± 0.07 logMAR at 1 week to 0.09 ± 0.08 logMAR at 3 months post-surgery. Similarly, the BCDVA improved marginally from 0.02 ± 0.05 logMAR at 1 week to 0.01 ± 0.03 logMAR at 3 months. For intermediate vision, UCIVA and DCIVA remained relatively stable, both recording a slight increase from 0.24 ± 0.10 logMAR at 1 week to 0.25 ± 0.09 and 0.25 ± 0.11 logMAR at 3 months, respectively. Near visual acuity measurements, both uncorrected (UCNVA) and distance-corrected (DCNVA), also showed slight but nonsignificant changes from 0.24 ± 0.11 and 0.21 ± 0.12 logMAR at 1 week to 0.26 ± 0.08 and 0.24 ± 0.09 logMAR at 3 months, respectively.

**Table 1:** Comparison of postoperative visual acuity (logMAR) at 1 wk and 3 mo after multifocal intraocular lens implantation.

Time	Uncorrected distance visual acuity	Best-corrected distance visual acuity	Uncorrected intermediate visual acuity	Distance-corrected intermediate visual acuity	Uncorrected near visual acuity	Distance-corrected near visual acuity
1 wk after surgery	0.06 ± 0.07	0.02 ± 0.05	0.24 ± 0.10	0.24 ± 0.10	0.24 ± 0.11	0.21 ± 0.12
3 mo after surgery	0.09 ± 0.08	0.01 ± 0.03	0.25 ± 0.09	0.25 ± 0.11	0.26 ± 0.08	0.24 ± 0.09
*Z* value	−1.052	−0.688	−0.06	−0.058	−0.672	−1.103
*P* value	.272	.445	.903	.954	.456	.234

Data were analyzed using the Mann–Whitney *U* test for non-normally distributed continuous variables.

Despite these minor fluctuations, the *Z* values derived from the Mann–Whitney *U* test for non-normally distributed continuous variables revealed no statistically significant changes over the examined period, reinforcing the stability of visual acuity outcomes post-surgery under the conditions tested.

### 3.2. Comparison of scatter light values under 3 and 5 mm apertures

As presented in Table 2, at the 3 mm aperture, there were no significant differences in scatter light values (log [*s*]) between 1 week and third months after surgery (*P* > .05). Conversely, under the 5 mm aperture, there was a significant decrease in scatter light values (log [*s*]) at the third month postoperatively compared to the first week (*P* < .05). Furthermore, the scattered light values under the 5 mm aperture were significantly larger than those under the 3 mm aperture at both 1 week and 3 months postoperatively (all *P *< .05), suggesting more pronounced light scattering under larger pupil conditions.

**Table 2:** Comparison of postoperative straylight values (log [*s*]) at 3 and 5 mm apertures at 1 week and 3 months.

Time	3 mm straylight values (log [*s*])	5 mm straylight values (log [*s*])	*Z* value	*P* value
1 wk after surgery	0.88 ± 0.15	1.05 ± 0.16	−3.658	<.001
3 mo after surgery	0.82 ± 0.09	0.95 ± 0.12	−4.516	<.001
*Z* value	−1.520	−1.913	–	–
*P* value	.128	.036	–	–

Differences in straylight values were assessed using the Mann–Whitney *U* test.

### 3.3. Root mean square (RMS) values of total eye aberrations under 3 and 5 mm apertures

As illustrated in Table 3, under the 3 mm aperture, the RMS value of trefoil aberrations at 3 months postoperatively was significantly lower than that at 1 week postoperatively (*P* < .05). Under the 5 mm aperture, the RMS value of spherical aberrations at 3 months postoperatively was also significantly lower than that at 1 week postoperatively (*P* < .05), implying a notable improvement in these specific aberrations over time.

**Table 3:** Comparison of wavefront aberration root mean square (RMS) values under 3 and 5 mm pupil sizes at 1 week and 3 months after surgery.

Time	3 mm pupil size (μm)					5 mm pupil size (μm)				
	Total	High	Spherical	Coma	Trefoil	Total	High	Spherical	Coma	Trefoil
1 wk after surgery	0.42 ± 0.13	0.20 ± 0.09	−0.02 ± 0.03	0.11 ± 0.09	0.17 ± 0.20	1.14 ± 0.42	0.53 ± 0.28	−0.07 ± 0.13	0.26 ± 0.16	0.31 ± 0.17
3 months after surgery	0.44 ± 0.16	0.15 ± 0.07	0.00 ± 0.04	0.07 ± 0.05	0.09 ± 0.06	1.16 ± 0.48	0.44 ± 0.14	−0.01 ± 0.05	0.18 ± 0.07	0.25 ± 0.14
*Z* value	−0.65205	−2.01915	−0.777	−1.42905	−2.0811	−0.1869	−1.55295	−3.3852	−1.5225	−1.9257
*P* value	.5607	.05775	.48195	.1827	.04935	.90195	.14595	.00105	0.15435	0.07035

Wavefront aberration comparisons were conducted using nonparametric tests due to non-normal distributions as confirmed by the Shapiro–Wilk test.

### 3.4. MTF values at different spatial frequencies under 3 and 5 mm apertures

As shown in Table 4, under the 3 mm aperture, a statistically significant difference was found in the MTF values at the high spatial frequency of 25 cycles per degree (cpd) between the first and third months postoperatively (*P* < .05). In contrast, under the 5 mm aperture, the MTF values at all spatial frequencies at 3 months postoperatively were higher than those at 1 week postoperatively. This difference was particularly significant at the intermediate spatial frequency of 20 cpd (*P* < .05), suggesting an improvement in contrast sensitivity with time and under different pupil conditions.

**Table 4:** Comparative analysis of modulation transfer function (MTF) values at 3 and 5 mm pupil sizes following multifocal intraocular lens implantation surgery.

Time Interval	MTF with 3 mm pupil size						MTF with 5 mm Pupil Size					
	5 cpd	10 cpd	15 cpd	20 cpd	25 cpd	30 cpd	5 cpd	10 cpd	15 cpd	20 cpd	25 cpd	30 cpd
1 wk after surgery	0.65 ± 0.21	0.31 ± 0.20	0.18 ± 0.13	0.11 ± 0.08	0.09 ± 0.08	0.11 ± 0.10	0.38 ± 0.16	0.13 ± 0.07	0.18 ± 0.13	0.06 ± 0.04	0.03 ± 0.02	0.03 ± 0.03
3 mo after surgery	0.72 ± 0.21	0.42 ± 0.26	0.28 ± 0.21	0.18 ± 0.19	0.14 ± 0.16	0.15 ± 0.15	0.47 ± 0.18	0.21 ± 0.12	0.28 ± 0.21	0.07 ± 0.05	0.06 ± 0.04	0.04 ± 0.03
*Z* value	−0.111	−0.023	−0.954	−1.442	−1.509	−0.888	−0.911	−0.821	−0.843	−1.665	−1.178	−0.267
*P* value	.662	.732	.152	.041	.033	.178	.169	.206	.196	.02	.087	.542

MTF values were evaluated using nonparametric tests for differences between 2 independent samples.

cpd = cycles per degree, MTF = modulation transfer function.

## 4. Discussion

The results of this study highlight the clinical benefits of Tecnis ZMB00 multifocal intraocular lenses (MIOLs) for enhancing visual acuity and reducing visual aberrations postcataract surgery across various pupil sizes. Significant reductions were observed in scatter light and higher-order aberrations, particularly spherical aberrations, which are crucial for optimizing night vision and minimizing halos and glare. These improvements were evident with marked enhancements in MTF values, particularly at intermediate spatial frequencies of 20 cycles per degree, improving contrast sensitivity and thereby enhancing patient quality of life for both daytime and nighttime visual functions. This is especially relevant for patients with active lifestyles who require superior intermediate and distance vision. Furthermore, the study documented a decrease in the RMS values of higher-order aberrations, including coma and trefoil, at both 3 and 5 mm apertures 3 months postoperatively, suggesting an interrelated reduction among various aberration parameters. Our findings also challenge the effectiveness of Zernike polynomials in clinical settings, as they do not accurately reflect the relative impact of each aberration. Notably, there was a statistically significant improvement in MTF values at a high spatial frequency of 25 cycles per degree under a 3 mm aperture, and at all tested spatial frequencies under a 5 mm aperture 3 months post-surgery, indicating a substantial enhancement in visual clarity and a reliable indicator of improved optical system performance. These outcomes support the continued use of Tecnis ZMB00 MIOLs in modern cataract surgery, aligning with the priorities of patient satisfaction and optimal vision quality.

In an ideal optical system, scattered light is absent as light perfectly focuses onto the retina.^[8,9]^ However, due to the human eye’s suboptimal anatomical structure, scattered light occurs, dispersing some light within the optical media and affecting the contrast of retinal imaging, which may cause visual disturbances such as halos and glare.^[10,11]^ This phenomenon is significant in assessing visual clarity post-pseudophakic surgery.^[12]^ Post-phacoemulsification, changes like a decrease in endothelial cell density and early corneal edema, particularly at the incision site, are noted.^[13]^ Corneal thickness typically stabilizes within 3 months post-surgery, which our study monitored.^[14]^ Our results indicated that while scattered light measured through a 3 mm aperture did not show a significant difference 1 week to 3 months post-surgery, the reduction in scattered light through a 5 mm aperture at 3 months was statistically significant, possibly linked to residual changes in corneal thickness near the incision. Additionally, macular changes post-surgery could influence scattered light levels, as indicated by the transient increase in central foseal thickness observed up to 6 weeks postoperatively.^[15]^ The Tecnis ZMB00 MIOL, designed with a −0.27 μm negative spherical aberration, effectively neutralizes the positive spherical aberration typically increasing with age in the human lens.^[16,17]^ Our findings confirmed a significant reduction in RMS values of spherical aberration 3 months post-surgery, aligning with the lens’s intended effect. This suggests that while macular thickness may influence scattered light, corneal adjustments post-surgery play a more significant role in visual outcomes following cataract surgery.

Recent publications have provided an in-depth understanding of postcataract surgery visual quality and its relationship with different types of intraocular lenses and techniques. Zhong et al^[18]^ highlighted FLACS’s superiority over CPCS in aberrations and optical quality under a 5.0 mm pupil diameter. In contrast, our research delves into postoperative visual quality with Tecnis ZMB00 MIOL implantation, emphasizing the influence of aperture conditions on outcomes. Both underscore pupil diameter’s role in evaluations, but our study offers nuanced insights into scatter light, aberrations, and MTF values at a 5 mm aperture, deepening MIOL performance understanding. Akpolat et al^[19]^ showcased improved vision-related quality-of-life post-phacoemulsification using NEI-VFQ-25 and FIM tests. Similarly, we highlight the benefits of cataract phacoemulsification with Tecnis ZMB00 MIOL, presenting a granular analysis of visual outcomes under distinct pupil conditions. Gallenga et al^[20]^ compared Acriva Reviol Tri-ED to monofocal IOLs, noting advantages in vision and aberration. Our study, focusing on Tecnis ZMB00 MIOL, reiterates the merits of advanced IOL technologies, presenting a meticulous exploration of visual outcomes under varied apertures. Narayan et al^[21]^ equated FLACS and PCS in efficacy, noting cost-effectiveness concerns with FLACS. Our study, centered on Tecnis ZMB00 MIOL postcataract phacoemulsification, enriches the discourse by emphasizing detailed postoperative visual and optical findings under specific aperture conditions.

Building on this foundation, our investigation into the Tecnis ZMB00 MIOL, a diffractive aspherical multifocal intraocular lens, reveals its significant clinical benefits. By employing a +4.0 D near add power, the lens mimics the effect of +3.2 D glasses, enhancing close vision. We evaluated how different pupil sizes influence visual acuity in various lighting conditions, using 2 aperture settings (3 and 5 mm). This study monitored 135 eyes over 3 months post, confirming that both uncorrected (UCDVA) and best-corrected (BCDVA) distance visual acuities remain stable. The lens’s design, which distributes light evenly between near and far focal points, often results in satisfactory intermediate vision, making it an excellent option for cataract patients seeking comprehensive visual improvement.

Our study, centered on Tecnis ZMB00 MIOL implantation, comes with distinct constraints. Although our sample size is relatively ample, the demographics remain narrow, which may influence result applicability. Despite using advanced tools, inherent measurement variabilities exist. The exclusive reliance on 1 surgeon and center could introduce specific biases. With evaluations at only 2 postoperative intervals, we might miss a fuller picture of recovery. Lacking a control group and the study’s retrospective nature further shape the insights we glean. Consequently, while our findings provide valuable perspectives, they call for judicious interpretation and broader subsequent studies.

Future research should address the limitations of this study by expanding participant diversity to improve the generalizability of results. Studies involving multiple surgeons and centers, as well as randomized controlled trial designs, are recommended to reduce biases and provide a more robust assessment of Tecnis ZMB00 MIOLs versus other intraocular lenses. Extending follow-up periods beyond 3 months is critical to evaluate long-term outcomes and late postoperative complications. Additionally, including functional vision assessments like quality-of-life measures and night driving simulations will better reflect the practical impact of these lenses on daily activities. It is also essential to consider that the higher-order aberrations and MTF values reported involve contributions from both corneal and internal ocular components, with changes in the corneal properties during the postoperative period not being independently assessed, which could influence the interpretation of these metrics. Future studies should isolate corneal aberrations to more accurately determine their impact separate from the intraocular lens effects. Moreover, further research should thoroughly examine the interactions between pupil size, visual acuity, and other optical quality metrics under controlled conditions, using advanced imaging techniques. Such investigations could identify the necessary levels of optical quality improvements for significant changes in visual acuity under realistic conditions, aiding in the optimal design and use of multifocal lenses for diverse patient groups in varying lighting conditions.

## 5. Conclusions

In conclusion, cataract extraction using phacoemulsification in conjunction with Tecnis ZMB00 MIOL implantation can provide patients with good distance, intermediate, and near visual acuity. Notably, at a pupil diameter of 5 mm, compared to 1-week post-surgery, 3 months post-surgery resulted in lower scatter light values, lower higher-order aberrations (particularly spherical aberration), and increased MTF values. Therefore, after cataract extraction using phacoemulsification and the implantation of Tecnis ZMB00 MIOL, not only is stable visual acuity and visual quality achieved, but the quality of night vision also improves to a certain extent.

## Author contributions

**Conceptualization:** Daoguang Wang.

**Data curation:** Daoguang Wang, Yuanxiao Ma, Yucheng Wang.

**Investigation:** Daoguang Wang.

**Methodology:** Daoguang Wang, Yuanxiao Ma, Tingting Hu.

**Writing – original draft:** Daoguang Wang, Yuanxiao Ma, Tingting Hu, Yucheng Wang.

**Formal analysis:** Yuanxiao Ma, Yucheng Wang.

**Resources:** Tingting Hu.

**Supervision:** Keli Cai.

**Writing – review & editing:** Keli Cai.
